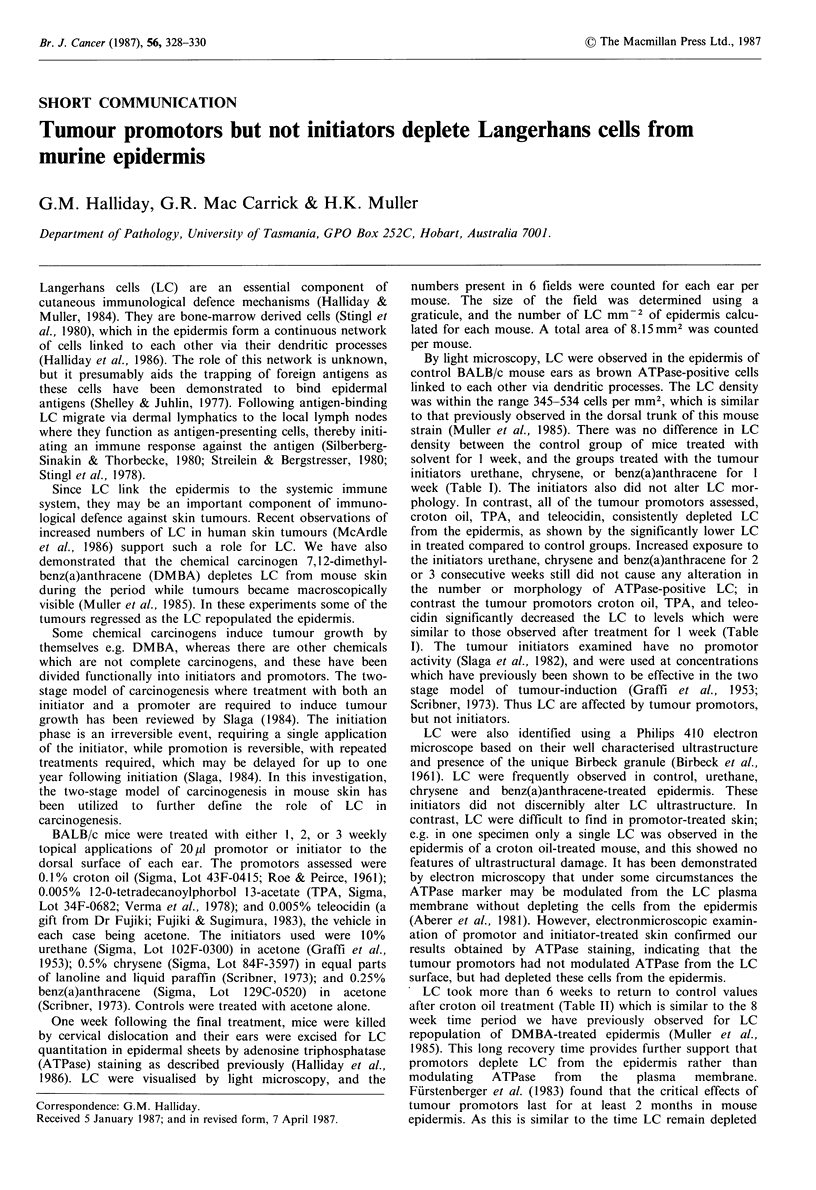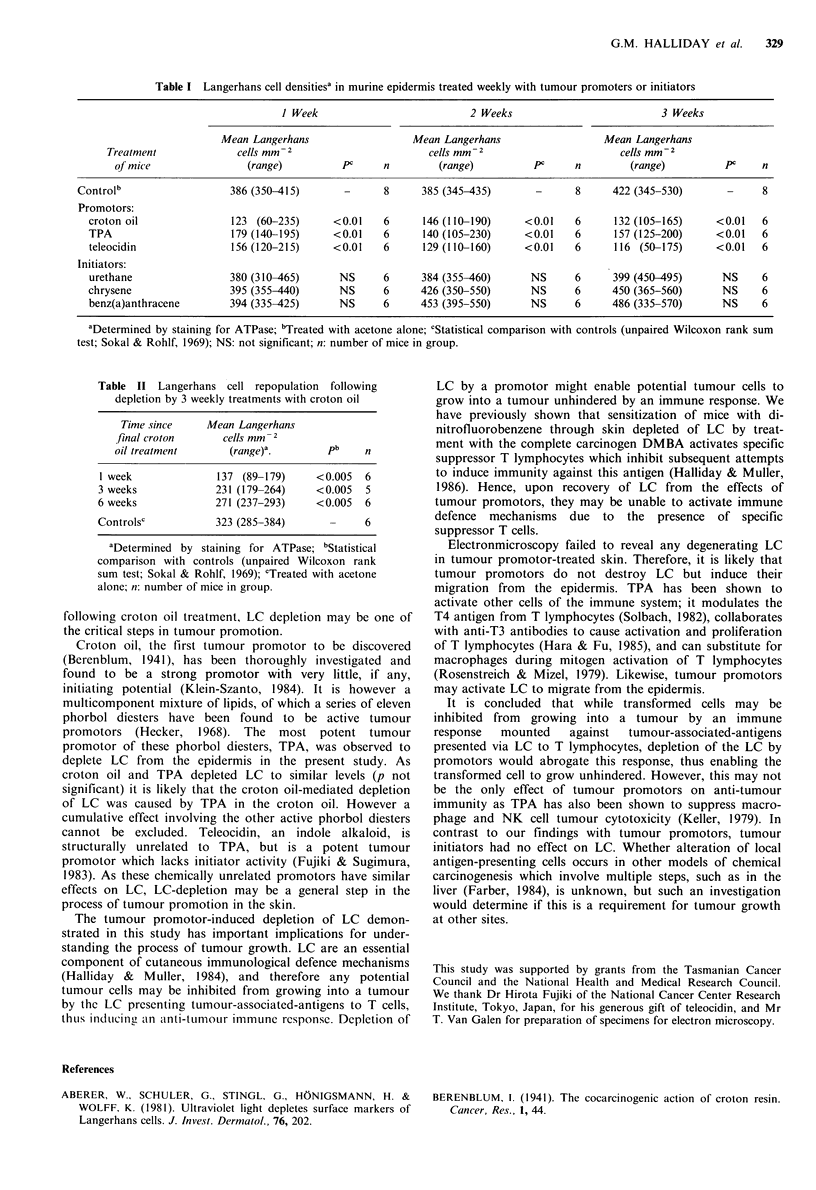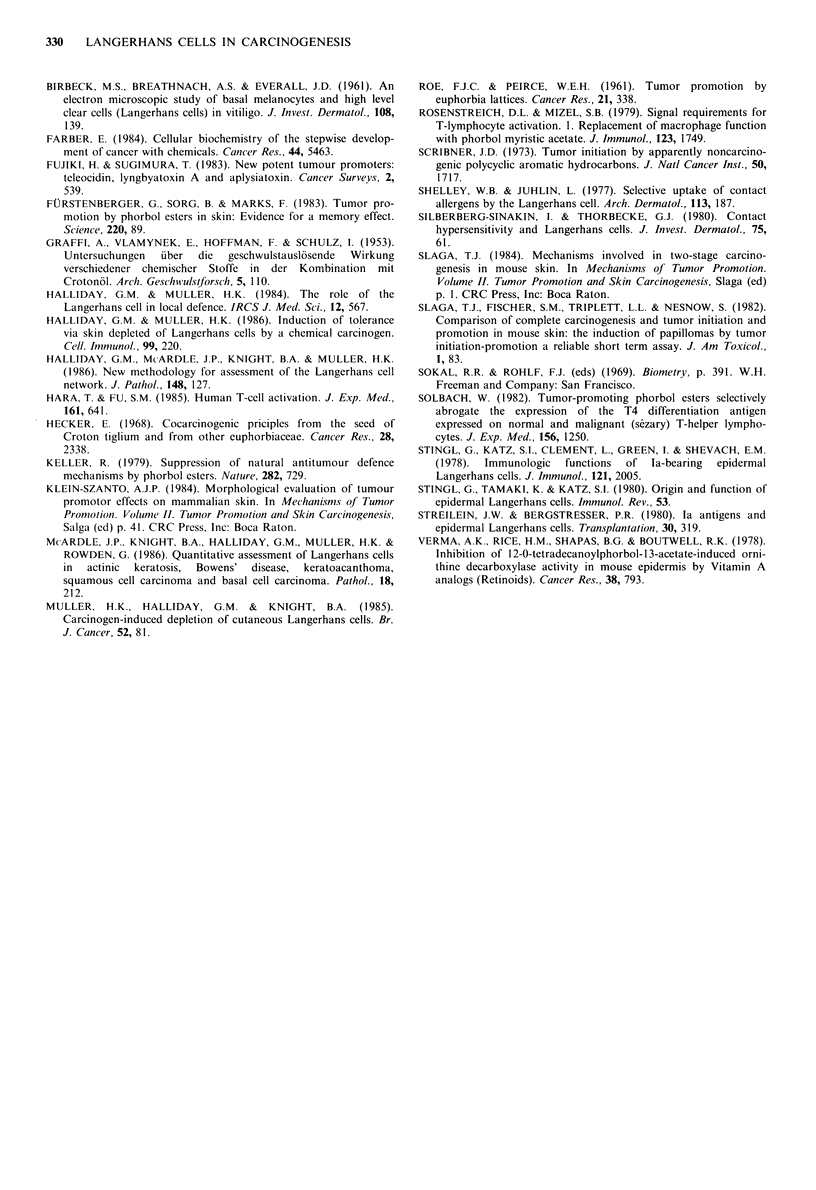# Tumour promoters but not initiators deplete Langerhans cells from murine epidermis.

**DOI:** 10.1038/bjc.1987.198

**Published:** 1987-09

**Authors:** G. M. Halliday, G. R. MacCarrick, H. K. Muller

**Affiliations:** Department of Pathology, University of Tasmania, Hobart, Australia.


*Br. J. Cancer* (1987), **56**, 328–330 © The Macmillan Press Ltd., 1987

SHORT COMMUNICATION

### Tumour promotors but not initiators deplete Langerhans cells from murine epidermis

G.M. Halliday, G.R. Mac Carrick & H.K. Muller

*Department of Pathology, University of Tasmania, GPO Box 252C, Hobart, Australia 7001.*

Langerhans cells (LC) are an essential component of cutaneous immunological defence mechanisms (Halliday & Muller, 1984). They are bone-marrow derived cells (Stingl *et al.*, 1980), which in the epidermis form a continuous network of cells linked to each other via their dendritic processes (Halliday *et al.*, 1986). The role of this network is unknown, but it presumably aids the trapping of foreign antigens as these cells have been demonstrated to bind epidermal antigens (Shelley & Juhlin, 1977). Following antigen-binding LC migrate via dermal lymphatics to the local lymph nodes where they function as antigen-presenting cells, thereby initiating an immune response against the antigen (Silberberg-Sinakin & Thorbecke, 1980; Streilein & Bergstresser, 1980; Stingl *et al.*, 1978).

Since LC link the epidermis to the systemic immune system, they may be an important component of immunological defence against skin tumours. Recent observations of increased numbers of LC in human skin tumours (McArdle *et al.*, 1986) support such a role for LC. We have also demonstrated that the chemical carcinogen 7,12-dimethylbenz(a)anthracene (DMBA) depletes LC from mouse skin during the period while tumours became macroscopically visible (Muller *et al.*, 1985). In these experiments some of the tumours regressed as the LC repopulated the epidermis.

Some chemical carcinogens induce tumour growth by themselves e.g. DMBA, whereas there are other chemicals which are not complete carcinogens, and these have been divided functionally into initiators and promotors. The two-stage model of carcinogenesis where treatment with both an initiator and a promoter are required to induce tumour growth has been reviewed by Slaga (1984). The initiation phase is an irreversible event, requiring a single application of the initiator, while promotion is reversible, with repeated treatments required, which may be delayed for up to one year following initiation (Slaga, 1984). In this investigation, the two-stage model of carcinogenesis in mouse skin has been utilized to further define the role of LC in carcinogenesis.

BALB/c mice were treated with either 1, 2, or 3 weekly topical applications of 20 $\mu$l promotor or initiator to the dorsal surface of each ear. The promotors assessed were 0.1% croton oil (Sigma, Lot 43F-0415; Roe & Peirce, 1961); 0.005% 12-0-tetradecanoylphorbol 13-acetate (TPA, Sigma, Lot 34F-0682; Verma *et al.*, 1978); and 0.005% teleocidin (a gift from Dr Fujiki; Fujiki & Sugimura, 1983), the vehicle in each case being acetone. The initiators used were 10% urethane (Sigma, Lot 102F-0300) in acetone (Graffi *et al.*, 1953); 0.5% chrysene (Sigma, Lot 84F-3597) in equal parts of lanoline and liquid paraffin (Scribner, 1973); and 0.25% benz(a)anthracene (Sigma, Lot 129C-0520) in acetone (Scribner, 1973). Controls were treated with acetone alone.

One week following the final treatment, mice were killed by cervical dislocation and their ears were excised for LC quantitation in epidermal sheets by adenosine triphosphatase (ATPase) staining as described previously (Halliday *et al.*, 1986). LC were visualised by light microscopy, and the numbers present in 6 fields were counted for each ear per mouse. The size of the field was determined using a graticule, and the number of LC $mm^{-2}$ of epidermis calculated for each mouse. A total area of 8.15 $mm^2$ was counted per mouse.

By light microscopy, LC were observed in the epidermis of control BALB/c mouse ears as brown ATPase-positive cells linked to each other via dendritic processes. The LC density was within the range 345–534 cells per $mm^2$, which is similar to that previously observed in the dorsal trunk of this mouse strain (Muller *et al.*, 1985). There was no difference in LC density between the control group of mice treated with solvent for 1 week, and the groups treated with the tumour initiators urethane, chrysene, or benz(a)anthracene for 1 week (Table I). The initiators also did not alter LC morphology. In contrast, all of the tumour promotors assessed, croton oil, TPA, and teleocidin, consistently depleted LC from the epidermis, as shown by the significantly lower LC in treated compared to control groups. Increased exposure to the initiators urethane, chrysene and benz(a)anthracene for 2 or 3 consecutive weeks still did not cause any alteration in the number or morphology of ATPase-positive LC; in contrast the tumour promotors croton oil, TPA, and teleocidin significantly decreased the LC to levels which were similar to those observed after treatment for 1 week (Table I). The tumour initiators examined have no promotor activity (Slaga *et al.*, 1982), and were used at concentrations which have previously been shown to be effective in the two stage model of tumour-induction (Graffi *et al.*, 1953; Scribner, 1973). Thus LC are affected by tumour promotors, but not initiators.

LC were also identified using a Philips 410 electron microscope based on their well characterised ultrastructure and presence of the unique Birbeck granule (Birbeck *et al.*, 1961). LC were frequently observed in control, urethane, chrysene and benz(a)anthracene-treated epidermis. These initiators did not discernibly alter LC ultrastructure. In contrast, LC were difficult to find in promotor-treated skin; e.g. in one specimen only a single LC was observed in the epidermis of a croton oil-treated mouse, and this showed no features of ultrastructural damage. It has been demonstrated by electron microscopy that under some circumstances the ATPase marker may be modulated from the LC plasma membrane without depleting the cells from the epidermis (Aberer *et al.*, 1981). However, electronmicroscopic examination of promotor and initiator-treated skin confirmed our results obtained by ATPase staining, indicating that the tumour promotors had not modulated ATPase from the LC surface, but had depleted these cells from the epidermis.

LC took more than 6 weeks to return to control values after croton oil treatment (Table II) which is similar to the 8 week time period we have previously observed for LC repopulation of DMBA-treated epidermis (Muller *et al.*, 1985). This long recovery time provides further support that promotors deplete LC from the epidermis rather than modulating ATPase from the plasma membrane. Fürstenberger *et al.* (1983) found that the critical effects of tumour promotors last for at least 2 months in mouse epidermis. As this is similar to the time LC remain depleted

Correspondence: G.M. Halliday.
Received 5 January 1987; and in revised form, 7 April 1987.

**Table I** Langerhans cell densities[a] in murine epidermis treated weekly with tumour promoters or initiators

| Treatment of mice | 1 Week: Mean Langerhans cells $mm^{-2}$ (range) | $P^c$ | n | 2 Weeks: Mean Langerhans cells $mm^{-2}$ (range) | $P^c$ | n | 3 Weeks: Mean Langerhans cells $mm^{-2}$ (range) | $P^c$ | n |
|---|---|---|---|---|---|---|---|---|---|
| Control[b] | 386 (350–415) | – | 8 | 385 (345–435) | – | 8 | 422 (345–530) | – | 8 |
| Promotors: | | | | | | | | | |
| croton oil | 123 (60–235) | <0.01 | 6 | 146 (110–190) | <0.01 | 6 | 132 (105–165) | <0.01 | 6 |
| TPA | 179 (140–195) | <0.01 | 6 | 140 (105–230) | <0.01 | 6 | 157 (125–200) | <0.01 | 6 |
| teleocidin | 156 (120–215) | <0.01 | 6 | 129 (110–160) | <0.01 | 6 | 116 (50–175) | <0.01 | 6 |
| Initiators: | | | | | | | | | |
| urethane | 380 (310–465) | NS | 6 | 384 (355–460) | NS | 6 | 399 (450–495) | NS | 6 |
| chrysene | 395 (355–440) | NS | 6 | 426 (350–550) | NS | 6 | 450 (365–560) | NS | 6 |
| benz(a)anthracene | 394 (335–425) | NS | 6 | 453 (395–550) | NS | 6 | 486 (335–570) | NS | 6 |

[a]Determined by staining for ATPase; [b]Treated with acetone alone; [c]Statistical comparison with controls (unpaired Wilcoxon rank sum test; Sokal & Rohlf, 1969); NS: not significant; *n*: number of mice in group.

**Table II** Langerhans cell repopulation following depletion by 3 weekly treatments with croton oil

| Time since final croton oil treatment | Mean Langerhans cells $mm^{-2}$ (range)[a]. | $P^b$ | n |
|---|---|---|---|
| 1 week | 137 (89–179) | <0.005 | 6 |
| 3 weeks | 231 (179–264) | <0.005 | 5 |
| 6 weeks | 271 (237–293) | <0.005 | 6 |
| Controls[c] | 323 (285–384) | – | 6 |

[a]Determined by staining for ATPase; [b]Statistical comparison with controls (unpaired Wilcoxon rank sum test; Sokal & Rohlf, 1969); [c]Treated with acetone alone; *n*: number of mice in group.

following croton oil treatment, LC depletion may be one of the critical steps in tumour promotion.

Croton oil, the first tumour promotor to be discovered (Berenblum, 1941), has been thoroughly investigated and found to be a strong promotor with very little, if any, initiating potential (Klein-Szanto, 1984). It is however a multicomponent mixture of lipids, of which a series of eleven phorbol diesters have been found to be active tumour promotors (Hecker, 1968). The most potent tumour promotor of these phorbol diesters, TPA, was observed to deplete LC from the epidermis in the present study. As croton oil and TPA depleted LC to similar levels ($p$ not significant) it is likely that the croton oil-mediated depletion of LC was caused by TPA in the croton oil. However a cumulative effect involving the other active phorbol diesters cannot be excluded. Teleocidin, an indole alkaloid, is structurally unrelated to TPA, but is a potent tumour promotor which lacks initiator activity (Fujiki & Sugimura, 1983). As these chemically unrelated promotors have similar effects on LC, LC-depletion may be a general step in the process of tumour promotion in the skin.

The tumour promotor-induced depletion of LC demonstrated in this study has important implications for understanding the process of tumour growth. LC are an essential component of cutaneous immunological defence mechanisms (Halliday & Muller, 1984), and therefore any potential tumour cells may be inhibited from growing into a tumour by the LC presenting tumour-associated-antigens to T cells, thus inducing an anti-tumour immune response. Depletion of LC by a promotor might enable potential tumour cells to grow into a tumour unhindered by an immune response. We have previously shown that sensitization of mice with dinitrofluorobenzene through skin depleted of LC by treatment with the complete carcinogen DMBA activates specific suppressor T lymphocytes which inhibit subsequent attempts to induce immunity against this antigen (Halliday & Muller, 1986). Hence, upon recovery of LC from the effects of tumour promotors, they may be unable to activate immune defence mechanisms due to the presence of specific suppressor T cells.

Electronmicroscopy failed to reveal any degenerating LC in tumour promotor-treated skin. Therefore, it is likely that tumour promotors do not destroy LC but induce their migration from the epidermis. TPA has been shown to activate other cells of the immune system; it modulates the T4 antigen from T lymphocytes (Solbach, 1982), collaborates with anti-T3 antibodies to cause activation and proliferation of T lymphocytes (Hara & Fu, 1985), and can substitute for macrophages during mitogen activation of T lymphocytes (Rosenstreich & Mizel, 1979). Likewise, tumour promotors may activate LC to migrate from the epidermis.

It is concluded that while transformed cells may be inhibited from growing into a tumour by an immune response mounted against tumour-associated-antigens presented via LC to T lymphocytes, depletion of the LC by promotors would abrogate this response, thus enabling the transformed cell to grow unhindered. However, this may not be the only effect of tumour promotors on anti-tumour immunity as TPA has also been shown to suppress macrophage and NK cell tumour cytotoxicity (Keller, 1979). In contrast to our findings with tumour promotors, tumour initiators had no effect on LC. Whether alteration of local antigen-presenting cells occurs in other models of chemical carcinogenesis which involve multiple steps, such as in the liver (Farber, 1984), is unknown, but such an investigation would determine if this is a requirement for tumour growth at other sites.

This study was supported by grants from the Tasmanian Cancer Council and the National Health and Medical Research Council. We thank Dr Hirota Fujiki of the National Cancer Center Research Institute, Tokyo, Japan, for his generous gift of teleocidin, and Mr T. Van Galen for preparation of specimens for electron microscopy.

### References

ABERER, W., SCHULER, G., STINGL, G., HÖNIGSMANN, H. & WOLFF, K. (1981). Ultraviolet light depletes surface markers of Langerhans cells. *J. Invest. Dermatol.*, **76**, 202.

BERENBLUM, I. (1941). The cocarcinogenic action of croton resin. *Cancer, Res.*, **1**, 44.

BIRBECK, M.S., BREATHNACH, A.S. & EVERALL, J.D. (1961). An electron microscopic study of basal melanocytes and high level clear cells (Langerhans cells) in vitiligo. *J. Invest. Dermatol.*, **108**, 139.

FARBER, E. (1984). Cellular biochemistry of the stepwise development of cancer with chemicals. *Cancer Res.*, **44**, 5463.

FUJIKI, H. & SUGIMURA, T. (1983). New potent tumour promoters: teleocidin, lyngbyatoxin A and aplysiatoxin. *Cancer Surveys*, **2**, 539.

FÜRSTENBERGER, G., SORG, B. & MARKS, F. (1983). Tumor promotion by phorbol esters in skin: Evidence for a memory effect. *Science*, **220**, 89.

GRAFFI, A., VLAMYNEK, E., HOFFMAN, F. & SCHULZ, I. (1953). Untersuchungen über die geschwulstauslösende Wirkung verschiedener chemischer Stoffe in der Kombination mit Crotonöl. *Arch. Geschwulstforsch*, **5**, 110.

HALLIDAY, G.M. & MULLER, H.K. (1984). The role of the Langerhans cell in local defence. *IRCS J. Med. Sci.*, **12**, 567.

HALLIDAY, G.M. & MULLER, H.K. (1986). Induction of tolerance via skin depleted of Langerhans cells by a chemical carcinogen. *Cell. Immunol.*, **99**, 220.

HALLIDAY, G.M., McARDLE, J.P., KNIGHT, B.A. & MULLER, H.K. (1986). New methodology for assessment of the Langerhans cell network. *J. Pathol.*, **148**, 127.

HARA, T. & FU, S.M. (1985). Human T-cell activation. *J. Exp. Med.*, **161**, 641.

HECKER, E. (1968). Cocarcinogenic priciples from the seed of Croton tiglium and from other euphorbiaceae. *Cancer Res.*, **28**, 2338.

KELLER, R. (1979). Suppression of natural antitumour defence mechanisms by phorbol esters. *Nature*, **282**, 729.

KLEIN-SZANTO, A.J.P. (1984). Morphological evaluation of tumour promotor effects on mammalian skin. In *Mechanisms of Tumor Promotion. Volume II. Tumor Promotion and Skin Carcinogenesis*, Salga (ed) p. 41. CRC Press, Inc: Boca Raton.

McARDLE, J.P., KNIGHT, B.A., HALLIDAY, G.M., MULLER, H.K. & ROWDEN, G. (1986). Quantitative assessment of Langerhans cells in actinic keratosis, Bowens' disease, keratoacanthoma, squamous cell carcinoma and basal cell carcinoma. *Pathol.*, **18**, 212.

MULLER, H.K., HALLIDAY, G.M. & KNIGHT, B.A. (1985). Carcinogen-induced depletion of cutaneous Langerhans cells. *Br. J. Cancer*, **52**, 81.

ROE, F.J.C. & PEIRCE, W.E.H. (1961). Tumor promotion by euphorbia lattices. *Cancer Res.*, **21**, 338.

ROSENSTREICH, D.L. & MIZEL, S.B. (1979). Signal requirements for T-lymphocyte activation. 1. Replacement of macrophage function with phorbol myristic acetate. *J. Immunol.*, **123**, 1749.

SCRIBNER, J.D. (1973). Tumor initiation by apparently noncarcinogenic polycyclic aromatic hydrocarbons. *J. Natl Cancer Inst.*, **50**, 1717.

SHELLEY, W.B. & JUHLIN, L. (1977). Selective uptake of contact allergens by the Langerhans cell. *Arch. Dermatol.*, **113**, 187.

SILBERBERG-SINAKIN, I. & THORBECKE, G.J. (1980). Contact hypersensitivity and Langerhans cells. *J. Invest. Dermatol.*, **75**, 61.

SLAGA, T.J. (1984). Mechanisms involved in two-stage carcinogenesis in mouse skin. In *Mechanisms of Tumor Promotion. Volume II. Tumor Promotion and Skin Carcinogenesis*, Slaga (ed) p. 1. CRC Press, Inc: Boca Raton.

SLAGA, T.J., FISCHER, S.M., TRIPLETT, L.L. & NESNOW, S. (1982). Comparison of complete carcinogenesis and tumor initiation and promotion in mouse skin: the induction of papillomas by tumor initiation-promotion a reliable short term assay. *J. Am Toxicol.*, **1**, 83.

SOKAL, R.R. & ROHLF, F.J. (eds) (1969). *Biometry*, p. 391. W.H. Freeman and Company: San Francisco.

SOLBACH, W. (1982). Tumor-promoting phorbol esters selectively abrogate the expression of the T4 differentiation antigen expressed on normal and malignant (sézary) T-helper lymphocytes. *J. Exp. Med.*, **156**, 1250.

STINGL, G., KATZ, S.I., CLEMENT, L., GREEN, I. & SHEVACH, E.M. (1978). Immunologic functions of Ia-bearing epidermal Langerhans cells. *J. Immunol.*, **121**, 2005.

STINGL, G., TAMAKI, K. & KATZ, S.I. (1980). Origin and function of epidermal Langerhans cells. *Immunol. Rev.*, **53**.

STREILEIN, J.W. & BERGSTRESSER, P.R. (1980). Ia antigens and epidermal Langerhans cells. *Transplantation*, **30**, 319.

VERMA, A.K., RICE, H.M., SHAPAS, B.G. & BOUTWELL, R.K. (1978). Inhibition of 12-0-tetradecanoylphorbol-13-acetate-induced ornithine decarboxylase activity in mouse epidermis by Vitamin A analogs (Retinoids). *Cancer Res.*, **38**, 793.